# Study on the disease burden of patients with mucopolysaccharidosis type II in China

**DOI:** 10.1186/s13023-024-03432-2

**Published:** 2024-11-05

**Authors:** Ni Yuan, Min Li, Shan-Shan Wang, Hua-Xin Yu, Ya-Qun Wang, Fan-Yu Dong, Han-Xiang Chen, Sheng-Nan Duan, Ji Luo

**Affiliations:** 1 School of Public Health, Dalian Medical University, Dalian, China; 2 Innovative Drug Policy and Medical Insurance Research Center, Dalian Medical University, Dalian, China; 3 School of Public Health, Jiamusi University, Jiamusi, China; 4 Medical Insurance Department of The First Hospital of China Medical University, Shenyang, China

**Keywords:** Mucopolysaccharidosis type II, Burden of disease, Influencing factor

## Abstract

**Background:**

In this study, we investigated the patient population of Mucopolysaccharidosis type II (MPS II) in China, understood the basic situation, prevalence and diagnosis and treatment status of the patients, as well as the economic burden of the patients, and analyzed the influencing factors.

**Methods:**

A cross-sectional study focusing on patients with MPS II was conducted in China in 2023. Participants in the study were drawn from the Beijing Zhengyu Mucopolysaccharide Rare Disease Care Center, which is the only non-profit organization in mainland China registered with the civil affairs department that focuses on mucopolysaccharidosis. Data were collected through an online questionnaire, which included basic patient information, disease status, self-assessment of quality of life, diagnosis and treatment, as well as direct and indirect medical costs. The demographic and diagnosis and treatment profile of patients were analyzed by descriptive statistics. Furthermore, univariate and multiple linear regression were used to explore the economic burden and influencing factors of patients with MPS II.

**Results:**

The survival data of 145 patients were collected, the majority (98.62%) were male, and 78 were less than or equal to 10 years old. All patients were covered by medical insurance, mainly urban residents (135 cases). In terms of expenses, the 124 patients in the year before the survey incurred a total cost of about 14.7895 million yuan, and the direct economic burden accounted for 87.19%. Univariate analysis showed that age, number of hospitalizations, length of hospital stay, number of outpatient/emergency departments, hematopoietic stem cell transplantation (HSCT), and enzyme replacement therapy (ERT) were significantly associated with the economic burden of disease. Multiple regression analysis showed that the number of hospitalizations, days of hospitalization, number of outpatient/emergency departments and HSCT treatment were the main influencing factors.

**Conclusions:**

This study found that patients with MPS II were difficult to diagnose and easily misdiagnosed, their physical functions were impaired in many aspects. The existing treatment options are not sufficient in terms of economy and effectiveness, and there is also a lack of corresponding policy guarantees and support, which makes patients and their families have to face huge financial pressure.

## Introduction

Mucopolysaccharidosis type II (MPS II) is a rare disease. Rare diseases, also known as “orphan diseases”, may be defined in different countries and regions according to their specific circumstances and healthcare systems, usually based on the number of rare diseases, incidence or harm degree [1]. The Orphan Drug Act defines rare diseases as those affecting fewer than 200,000 people. The European Union defines rare diseases as chronic, progressive, and life-threatening diseases with a prevalence of less than 50 per 100,000. In Japan, rare diseases are defined as those with a total number of ≤ 50,000 patients or a prevalence of < 40/100,000 [2]. In 2021, the “China Rare Disease Definition Research Report 2021” was released, which defined rare diseases in China as “diseases with a newborn incidence of less than 1/10,000, a prevalence of less than 1/10,000, and a number of patients less than 140,000”. This definition provided important guidance and reference for the development of rare diseases in China [3]. At present, there are more than 7,000 known rare diseases in the world, and in China, the number of rare disease patients in China exceeds 20 million [4]. In September 2023, China’s National Health Commission, Ministry of Science and Technology, Ministry of Industry and Information Technology, and six other departments jointly issued the second batch of Rare Disease Catalog, together with the first batch of Rare Disease Catalog issued by the five departments in May 2018, currently the catalog has covered 207 rare diseases, MPS II is also included [5].

MPS II was first described by Dr. Charles Hunter in 1917 and is also widely known as Hunter Syndrome [6]. MPS II is an X-linked multisystem disorder characterized by glycosaminoglycan (GAG) accumulation, caused by a deficiency of iduronate-2-sulfatase (IDS) [7]. The global incidence of MPS II is 1:100,000–170,000 male births, based on genetic characteristics, and is common in males [8]. Asian countries have a higher reported incidence of MPS II compared to other types of MPS, and in Taiwan, the incidence of MPS II is approximately 2.05 in 100,000 male live births [9]. The main symptoms of MPS II patients range from the typical gross facial features and hepatosplenomegaly to skeletal, respiratory, auditory, cardiac, and central and peripheral nervous system dysfunction and become progressively more pronounced with advancing age, multiple organs in the body are damaged [10]. MPS II is characterized by mild and severe forms, the life expectancy also differs in the two forms, patients with the mild form reach adulthood, while patients affected by the severe form usually die within the first two decades of life [11]. Up to now, there is still no effective radical treatment for MPS II, and the main treatment methods include hematopoietic stem cell transplantation (HSCT), enzyme replacement therapy (ERT), symptomatic therapy, etc [12]. Among them, ERT is a direct solution to the enzyme deficiency problem, but it requires lifelong cyclic medication and is expensive; HSCT can provide a durable lifelong enzyme source with a single intervention and improve the symptoms of neurological progression, but there is a greater therapeutic risk; symptomatic treatment mainly relieves the general symptoms of the disease, temporarily solves the problem, and does not provide a cure; in recent years, domestic and foreign scholars have begun to pay attention to the study of the burden of disease for patients with rare diseases, but there are fewer studies on the burden of disease for MPS II. In recent years, scholars at home and abroad have begun to pay attention to research on the burden of disease in patients with rare diseases, but there are fewer studies on the burden of disease in MPS II, which mainly focus on clinical and pharmaceutical research. To summarize, this study was conducted to understand the survival status and disease economic burden of MPS II patients in China.

## Methods

### Aim

This study is dedicated to evaluating the economic burden of disease among MPS II patients in China, as well as identifying the key factors affecting the financial burden of disease, to provide accurate and detailed data to support policymakers, researchers, and other sectors of the community, to draw widespread attention to the MPS II patient population and to promote the implementation of proactive and favorable policy measures for rare diseases.

### Design and setting of the study

**Fig. 1:**
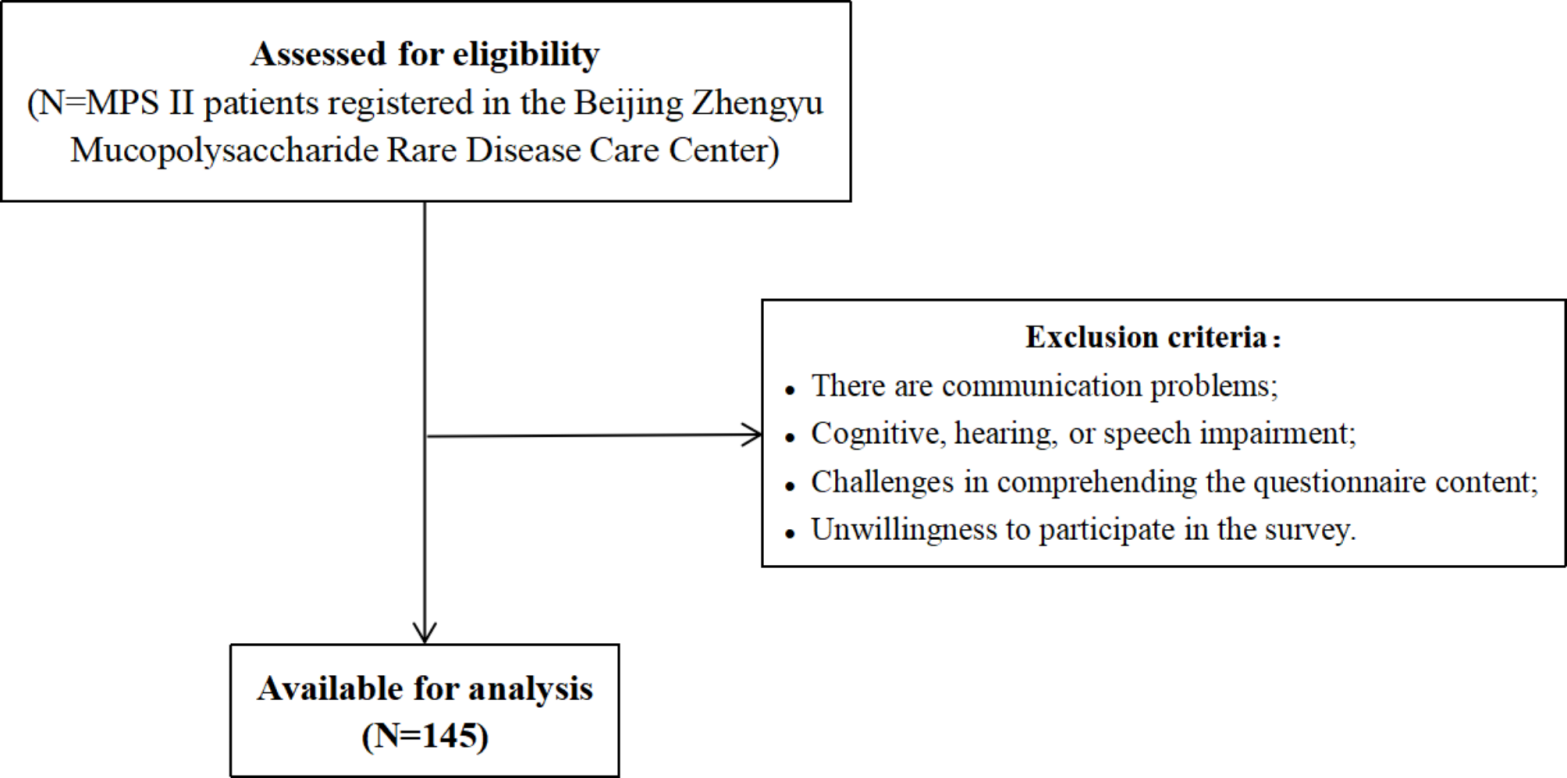
Flowchart of recruitment of the volunteers included in the study

At present, the sample size is generally determined based on a ratio of 5 to 10 times the number of analyzed variables. While the sample size in this study does not strictly adhere to the 10-times rule, it closely approximates it. Due to the particularity of rare diseases, the overall number of patients is minimal, and the platform for obtaining research is limited. A total of 145 patients’ survival survey data were collected in this study, and 0 invalid data were excluded (Fig. 1). This is the largest patient research project on the number of MPS II patients in China so far, and the sample size of this study is large compared to other studies on this disease.

The research constituted a cross-sectional study of MPS II patients within a specific demographic, initiated by the China Rare Disease Alliance, the subjects came from MPS II patients registered in the Beijing Zhengyu Mucopolysaccharide Rare Disease Care Center. The research group developed the questionnaire after fully understanding the current situation of MPS II through Chinese and foreign literature research, and after organizing consultation with patient experts and clinical experts. The questionnaire included patients’ socio-demographic characteristics, diagnosis and treatment experiences, and disease burden. Before the formal study, the research group conducted a pre-survey for proofreading the questionnaire for logic and rationality.

This study was mainly for the patients themselves, and if the patients were unable to complete the questionnaire, their guardians were authorized to complete it on their behalf. After obtaining informed consent, patients or their guardians can choose to participate in the survey or opt out directly. Before the patients or their guardians fill in the questionnaire, the trained investigators of the research group first introduce the purpose, process, rights and questionnaire to the patients in detail. The inclusion criteria of patients in this study were: (i) Patients clinically diagnosed as MPS II; (ii) Patients who voluntarily participate and sign informed consent. This study strictly followed the principles of ethics and informed consent and was approved by the Biomedical Ethics Committee of Dalian Medical University.

### Data and statistical analysis

The questionnaires were distributed through the cloud service platform of the CARRD after proofreading, and the original data were checked for logic and completeness after exporting the data from the cloud service platform. The collected patient data were cleaned using EXCEL software and then statistically analyzed using SPSS 26.0 software. Data analysis showed a skewed distribution of the patients’ disease economic burden. To accurately describe the average trend of disease economic burden, better reflect the overall economic burden level of the patient group, and avoid the influence of extreme values on the average, the median is chosen as the metric in this study. For comparisons between two independent samples, the Mann-Whitney U test was used in this study; the Kruskal-Wallis H test was used for comparisons between multiple independent samples. The inclusion of statistically significant independent variables in the multiple linear equations and the logarithmic transformation of the total costs resulted in an approximate normal distribution of data, which allowed for multiple linear regression analysis.

### Definition of relevant concepts

The burden of disease refers to the economic and health pressures of illness, disability, and premature death on patients, families, and society. Economic burden, on the other hand, is the direct and indirect impact of disease on the Society and Economic System and reflects the burden of disease on society.

- The direct economic burden mainly refers to the utilization of economic resources in medical institutions and other healthcare sectors. The direct financial burden in this study encompasses both direct medical expenses, such as out-of-pocket costs for MPS II visits/emergency room visits and hospitalizations in the year prior to the study, and direct non-medical expenses including transportation, food, accommodation, and other associated costs incurred by patients and their families during MPS II visits in the year prior to the study.
- Indirect economic burden refers to the reduction in effective working hours or capacity of the labor force due to illness, as well as the present value of lost productivity due to sickness-related absenteeism, loss of earnings, and loss of earnings due to early death. In this study, the indirect economic burden is defined as the productivity loss caused by MPS II absence of patients’ family members in the year before the survey began (number of non-full-time accompanying family members × average number of accompanying days per person × average income loss per person per day).

## Results

### Basic information about patients

#### Basic characteristics of patients

Out of 145 patients, 143 (98.62%) were male patients and 2 (1.38%) were female patients. In terms of age, the mean age of the investigated patients was (10.28 ± 5.95) years, 78 (53.79%) patients were ≤ 10 years old, of which the oldest patient was 35.26 years old, and the youngest was 1.83 years old. There were a total of 11 patients over the age of 18 years old, all of whom were unmarried and had no partner. The participation rate of the 145 patients in the national basic medical insurance was 100%, of which 10 (6.89%) patients were enrolled in urban residents’ basic health insurance and 135 (93.10%) patients were enrolled in urban workers’ basic medical insurance. In addition to basic medical insurance, 24 (16.55%) patients also participated in other social security or social assistance. In terms of annual family income, the mean value of patients’ total annual family income was (7.33 ± 7.96) ten thousand CNY, the median was 6.00 ten thousand CNY, and the maximum was 80.00 ten thousand CNY. 50 (34.48%) patients had an annual family income of less than 5 ten thousand CNY, and 95 (65.52%) patients had an annual family income of 5 ten thousand CNY and above (Table 1).

**Table 1:** Basic sociodemographic characteristics of 145 MPS II patients

Basic demographic characterization variables	No. of people	Ratios(%)
**Gender**		
Male	143	98.62
Female	2	1.38
**Age**		
≤ 10	78	53.79
10–20	59	40.69
> 20	8	5.52
**Type of health insurance**		
Urban employee basic medical insurance	10	6.89
Urban and rural residents’ basic medical insurance	135	93.10
**Whether you participate in other social security** **or receive social assistance**		
Yes	24	16.55
No	121	83.45
**Annual household income (ten thousand CNY)**		
< 5	50	34.48
≥ 5	95	65.52

#### Diagnosis and treatment of patients

**Fig. 2:**
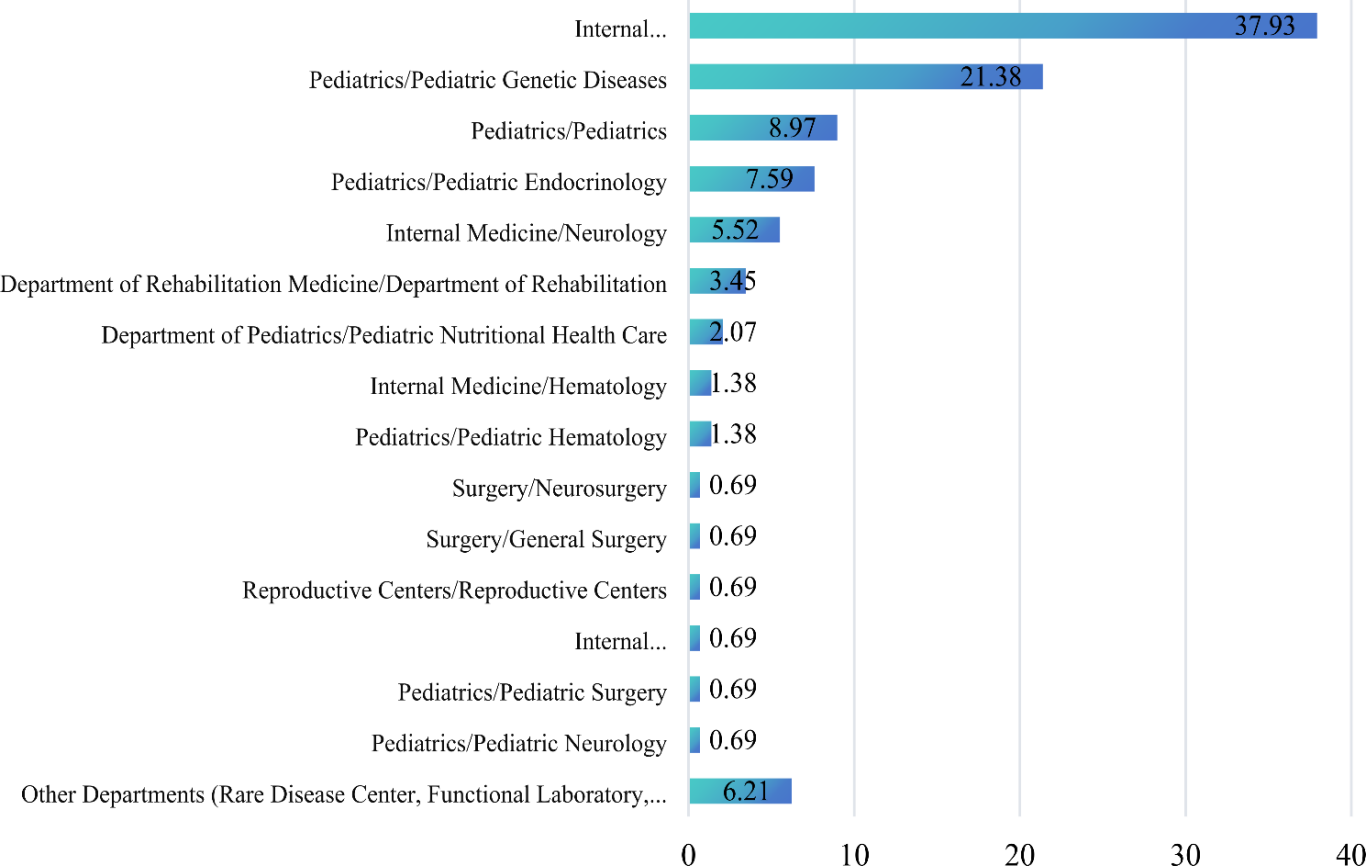
Department where the patient was diagnosed (%)

**Fig. 3:**
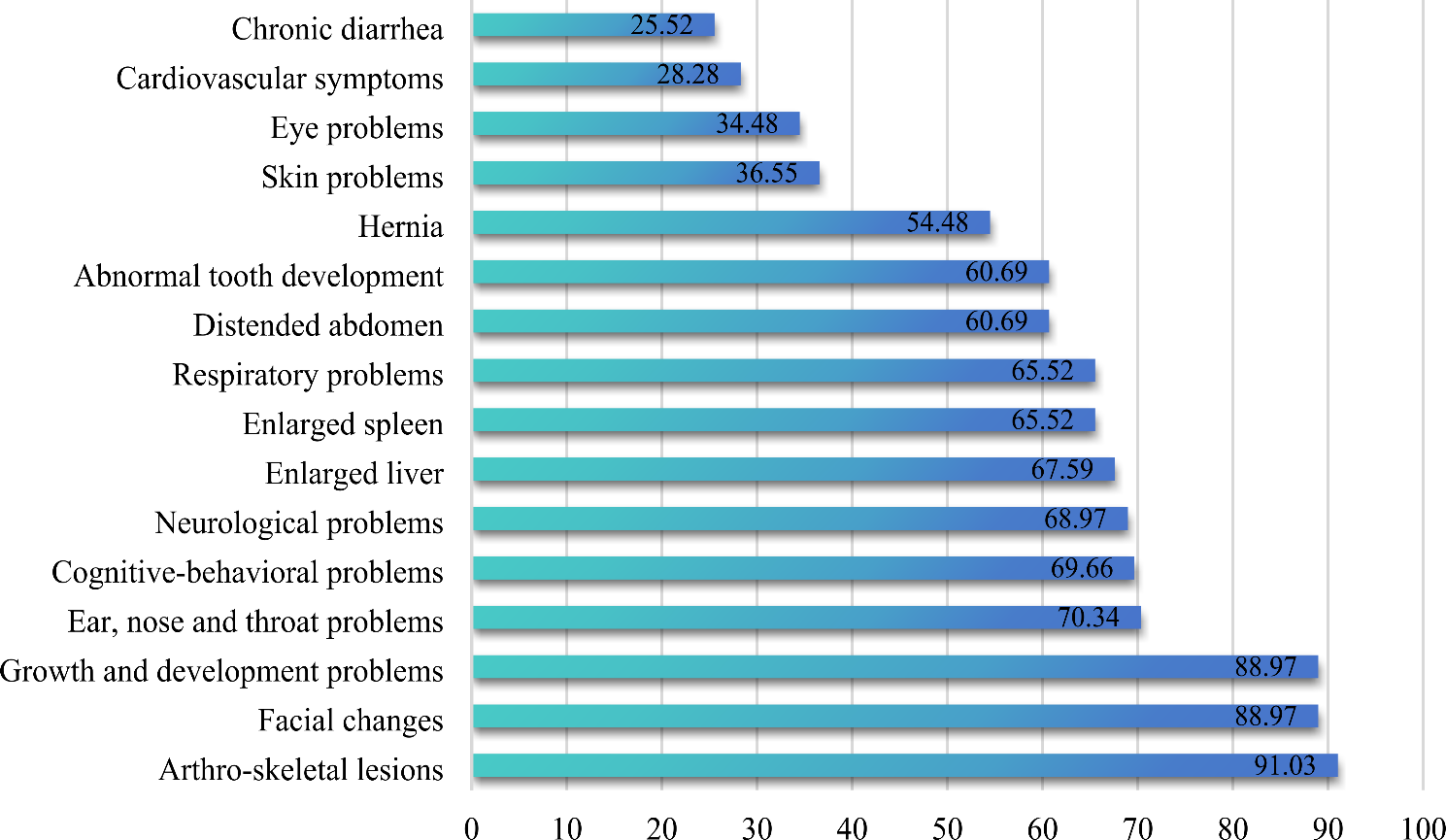
The proportion of main symptoms in patients (%)

According to the findings, 83 (57.24%) patients had experienced misdiagnosis. The results of the family prevalence survey showed that 109 (75.17%) patients did not have family members with the same disease. Before the birth of the patients, 88 (60.69%) patients were examined by both parents (Table 2). In terms of the department where the diagnosis was made, 37.93% of the patients were diagnosed in endocrinology and 21.38% were diagnosed in pediatric genetics (Fig. 2). The type of diagnosis made in 69 (47.59%) patients was severe, 39 (26.90%) patients were diagnosed in mild type and the rest were unknown. The main presenting symptoms of the patients had the highest percentage of arthro-skeletal lesions (91.03%), followed by growth and development problems (88.97%), and facial changes (88.97%) (Fig. 3).

During the period from first consultation to diagnosis, 80 (55.17%) patients had less than or equal to 3 hospital visits, and 65 (44.83%) patients had more than 3 hospital visits. In the past year, 60 (41.38%) patients had 1–5 outpatient/emergency visits, 67 (46.21%) patients had 0 hospitalizations, and 89 (61.38%) patients had ≤ 10 days of hospitalization. In terms of treatment received, 37 (25.52%) patients had received HSCT and 41 (28.28%) patients had received ERT (Table 2).

**Table 2:** Diagnosis and treatment of 145 MPS II patients

Disease characteristic variable	No. of people	Ratios (%)
**Have you ever experienced misdiagnosis**		
Yes	83	57.24
No	62	42.76
**Whether there are other family members** **with the same disease**		
Yes	36	24.83
No	109	75.17
**Types of patients at diagnosis**		
Mild	69	47.59
Severe	39	26.90
Unknown	37	25.52
**The number of tests performed at the time of diagnosis**		
≤ 3	71	48.97
> 3	74	51.03
**Number of hospital visits by patients from** **first visit to confirm of diagnosis**		
≤ 3	80	55.17
> 3	65	44.83
**Number of hospitalizations**		
0	67	46.21
1–5	50	34.48
> 5	28	18.31
**Patient hospitalization days**		
≤ 10	89	61.38
10–20	15	10.34
> 20	41	28.28
**Patient outpatient/emergency department visits**		
0	27	18.62
1–5	60	41.38
> 5	58	40.00
**Received HSCT or not**		
Yes	37	25.52
No	108	74.48
**Received ERT or not**		
Yes	41	28.28
No	104	71.72

### Economic burden of disease and composition of patients

Out of the 145 patients surveyed, 21 patients had no expenses, while 124 patients incurred costs for outpatient/emergency visits, hospitalizations, and other non-medical expenses in the year prior to the study. The total outpatient/emergency costs for these 124 patients amounted to approximately 400.18 ten thousand CNY, with a median of 1.00 (0.40, 3.00) ten thousand CNY. Additionally, the total hospitalization costs were about 541.66 ten thousand CNY, with a median of 0.63 (0.00, 4.43) ten thousand CNY. Furthermore, the total amount of direct non-medical expenses (including transportation costs, related food costs, accommodation costs, etc.) incurred by these patients was approximately 347.65 ten thousand CNY with a median of 2.00 (0.53, 3.38) ten thousand CNY; and their indirect cost was about 189.46 ten thousand CNY with a median of 0.07 (0.00, 0.81) ten thousand CNY.

The total economic burden of the 124 patients was about 1,478.95 ten thousand CNY, with a median of 5.38 (2.51, 12.14) ten thousand CNY. The total direct economic burden was 1,289.49 ten thousand CNY, and the median was 4.00 (2.03, 11.13) ten thousand CNY, accounting for 87.19% of the total disease economic burden. The total indirect economic burden was about 189.46 ten thousand CNY, and the median was 0.07 (0.00, 0.81) ten thousand CNY, accounting for 12.81% of the total disease economic burden. By comparing the direct medical expenses incurred by the patients with the total annual income of the family, the medical expenses incurred by the vast majority of patients due to MPS II exceed 40% of the total annual income of the family (catastrophic family health expenditure standard) [13] (Table 3).

**Table 3:** Total economic burden of disease of 124 patients (unit: ten thousand CNY)

Categories	Total cost(%)	Median (P25, P75)	Min value	Max value
**Direct economic burden**	1289.49(87.19)	4.00(2.03, 11.13)	0.00	78.80
Outpatient/emergency costs	400.18(31.03)	1.00(0.40, 3.00)	0.00	45.00
Hospitalization costs	541.66(42.01)	0.63(0.00, 4.43)	0.00	50.00
Direct non-medical costs	347.65(26.96)	2.00(0.53, 3.38)	0.00	20.00
**Indirect economic burden**	189.46(12.81)	0.07(0.00, 0.81)	0.00	28.00
**Total economic burden of disease**	1478.95(100.00)	5.38(2.51, 12.14)	0.11	78.80

### Analysis of influencing factors of disease economic burden

#### Monofactor analysis

For comparisons between two independent samples, the Mann-Whitney U test was used in this study, while for comparisons between multiple independent samples, the Kruskal-Wallis H test was used.

The results of the study showed that the age factor was statistically significant (*p* < 0.05) when comparing different demographic characteristics, which means that there are significant differences between different age groups that have an impact on the financial burden of disease. Among different ages, patients less than 10 years old had the highest median cost of 5.90 (2.97,19.40) ten thousand CNY, and the heaviest disease burden, followed by patients aged 10–20 years old with a median cost of 4.90 (2.52, 10.60) ten thousand CNY, and patients aged more than 20 years old with a minimum median cost of 2.05 (0.53, 3.23) ten thousand CNY. The differences were not statistically significant (*p* > 0.05) when comparing the financial burden of disease for demographic characteristics other than the age factor (Table 4).

**Table 4:** Comparative analysis of the economic burden of disease based on demographic characteristics of 124 patients (unit: ten thousand CNY)

Variables	No. of people(%)	Median (P25, P75)	Z value /H value	*P* value
**Gender**			-0.159	0.874
Male	122(98.39)	5.38(2.52, 12.00)		
Female	2(1.61)	10.15(1.00, 19.30)		
**Age**			6.905	0.032*
≤ 10	71(57.26)	5.90(2.97, 19.40)		
10–20	45(36.89)	4.90(2.52, 10.60)		
> 20	8(6.45)	2.05(0.53, 3.23)		
**Type of health insurance**			-0.174	0.283
Urban employee basic medical insurance	10(8.06)	10.41(2.75, 24.00)		
Urban and rural residents’ basic medical insurance	114(91.94)	5.19(2.50, 11.50)		
**Whether participating in other social security or** **receive social assistance**			-1.338	0.181
Yes	20(16.13)	8.81(3.18, 21.90)		
No	104(83.87)	4.97(2.40, 11.40)		
**Annual household income (ten thousand CNY)**			0.482	0.487
< 5	44(35.48)	4.88(2.79, 10.70)		
≥ 5	80(64.52)	6.55(2.30, 16.05)		

*P<0.05

According to the results of the diagnosis and treatment of patients, patients with the number of hospitalizations, hospitalization days, outpatient/emergency visits, whether they had received HSCT or not, and whether they had received ERT had statistical significance (*P* < 0.05). In the number of hospitalizations of patients, the median cost of patients hospitalized more than 5 times was the highest at 16.55 (9.09, 32.42) ten thousand CNY, followed by the median cost of patients hospitalized for 1–5 times was 8.50 (3.20, 16.00) ten thousand CNY, and the median cost of patients hospitalized for 0 times was the lowest 2.57 (1.00, 4.00) ten thousand CNY. Among the hospitalization days of patients, the median cost of patients with hospitalization days greater than 20 days was the highest at 16.20 (8.98, 35.70) ten thousand CNY, followed by the median cost of patients with hospitalization days 10–20 days was 8.79 (5.38, 16.00) ten thousand CNY. The lowest median cost for patients hospitalized for 10 days or less was 3.20 (1.50, 5.00) ten thousand CNY. From the perspective of the number of outpatient/emergency visits, the highest median cost of patients with more than 5 outpatient/emergency visits was 10.73 (5.38, 25.00) ten thousand CNY, followed by the median cost of patients with 1–5 outpatient/emergency visits was 3.70 (2.30, 8.83) ten thousand CNY. The minimum median cost for patients with 0 outpatient/emergency department visits was 1.00 (0.52, 2.00) ten thousand CNY.

According to the above results, there was a strong association between the patients with the number of hospitalizations, hospitalization days, and outpatient/emergency visits with the financial burden of the patient’s illness. The median cost of 11.50 (5.40, 34.05) ten thousand CNY for patients who had received HSCT was significantly higher than the median cost of 4.00 (2.00, 9.50) ten thousand CNY for patients who had not received HSCT. Due to the high cost of HSCT surgical treatment, patients may also need to take anti-rejection medications depending on whether they have a post-operative rejection reaction, creating a heavier financial burden of the disease. The median cost of patients who had received ERT was 9.76 (3.99, 22.90) ten thousand CNY, which was significantly higher than that of patients who had not received ERT, which was 4.28 (2.30, 10.31) ten thousand CNY. The economic burden of disease is higher for patients who have undergone ERT treatment due to the higher cost and long-term usage of the drugs, which are dependent on age and weight growth. There was no statistical significance found for other diagnoses, such as misdiagnosis experience, familial occurrence of the disease, or patient type at diagnosis (*P* > 0.05) (Table 5).

**Table 5:** Comparison of disease burden of 124 patients with different diagnosis and treatment conditions (unit: ten thousand CNY)

Variable	No. of people(%)	Median (P25, P75)	Min value	Max value
**Have you ever experienced misdiagnosis**			-0.03	0.098
Yes	74(59.68)	5.15(2.50, 14.00)		
No	50(40.32)	5.38(2.60, 10.85)		
**Whether there are other family members** **with the same disease**			-0.680	0.497
Yes	32(25.81)	5.75(2.79, 12.09)		
No	92(74.19)	5.38(2.30, 12.50)		
**Types of patients at diagnosis**			5.847	0.054
Mild	33(26.61)	3.95(2.25, 9.35)		
Severe	60(48.39)	6.50(2.60, 16.30)		
Unknown	31(25.00)	8.00(2.30, 16.10)		
**The number of tests performed at the** **time of diagnosis**			-1.611	0.107
≤ 3	59(47.58)	4.56(2.30, 10.60)		
> 3	65(52.42)	8.00(2.30, 16.10)		
**Number of hospital visits by patients from** **first visit to confirm the diagnosis**			-0.225	0.822
≤ 3	64(51.61)	5.55(2.30, 11.84)		
> 3	60(48.39)	5.38(2.59, 12.75)		
**Number of hospitalizations**			47.689	0.000*
0	47(37.90)	2.57(1.00, 4.00)		
1–5	49(39.52)	8.50(3.20, 16.00)		
> 5	28(22.58)	16.55(9.09, 32.42)		
**Patient hospitalization days**			51.732	0.000*
≤ 10	69(55.64)	3.20(1.50, 5.00)		
10–20	15(12.10)	8.79(5.38, 16.00)		
> 20	40(32.26)	16.20(8.98, 35.70)		
**Patient outpatient/emergency department visits**			37.621	0.000*
0	9(7.26)	1.00(0.52, 2.00)		
1–5	57(45.97)	3.70(2.30, 8.83)		
> 5	58(46.77)	10.73(5.38, 25.00)		
**Received HSCT or not**			-4.173	0.000*
Yes	33(26.61)	11.50(5.40, 34.05)		
No	91(73.39)	4.00(2.00, 9.50)		
**Received ERT or not**			-2.703	0.007*
Yes	36(29.03)	9.76(3.99, 22.90)		
No	88(70.97)	4.28(2.30, 10.31)		

*P<0.05

#### Multivariate analysis

The inclusion of statistically significant independent variables in the multiple linear equations and the logarithmic transformation of the total costs allowed for an approximate normal distribution of the data, which led to the multiple linear regression analysis. The results showed that the patients the number of hospitalizations, hospitalization days, outpatient/emergency visits, and whether they had received HSCT or not were the main factors affecting the economic burden of the disease (*P* < 0.05). Patients face a heavier financial burden as a result of increased hospitalizations, longer days in the hospital, and more outpatient/emergency visits. In addition, patients who had received HSCT also bore higher costs (Tables 6 and 7). According to the hypothesis test of the regression model, F = 26.221, *p* < 0.001, indicating that the established regression model is statistically significant. The multiple linear regression equation with R^2^ = 0.573 indicates that 57.3% of the variation in Y_1_ (Lg economic burden of disease) can be explained by the following equation with goodness of fit. By including factors with significant effects in the regression equation, by including factors with significant effects, we have developed a regression equation on the economic burden of disease. It is shown below:

1 $$\:{\text{Y}}_{1}=-0.324+0.174{\text{X}}_{2}+0.193{\text{X}}_{3}+0.270{\text{X}}_{4}+0.234{\text{X}}_{5}$$

**Table 6:** Quantitative assignments for multiple linear regressions of the economic burden of disease

Variable Name	Code	Assignment method
Lg The economic burden of disease	Y_1_	Continuous variable
Age	X_1_	1 = ≤ 10 years 2 = 10–20 years 3 = > 20 years
Number of hospitalizations	X_2_	1 = 0 times 2 = 1–5 times 3 = > 5 times
Patient hospitalization days	X_3_	1 = ≤ 10 years 2 = 10–20 years 3 = > 20 years
Patient outpatient/emergency department visits	X_4_	1 = 0 times 2 = 1–5 times 3 = > 5 times
Received HSCT or not	X_5_	1 = Yes 2 = No
Received ERT or not	X_6_	1 = Yes 2 = No

**Table 7:** Multiple linear regression results of factors influencing patients’ burden of disease

Independent variable	Non-standard conversion coefficient		Standard coefficient	T	*P*
	Regression coefficient	Standard error			
(Constant)	-0.324	0.181		-1.795	0.075
X_2_Number of Hospitalizations	0.174	0.068	0.226	2.536	0.013
X_3_Patient hospitalization days	0.193	0.056	0.299	3.413	0.001
X_4_Patient Outpatient/emergency department visits	0.270	0.065	0.286	4.175	0.000
X_5_Received HSCT or not	0.234	0.095	0.177	2.457	0.015

## Discussion

In this study, it was determined that MPS II primarily develops between the ages of 0 and 10, presenting as skeletal deformities, developmental delays, and multi-organ involvement. Early diagnosis and prompt treatment are crucial for MPS II patients, as a timely intervention in the early stages of the disease can prevent or delay its progression and mitigate serious complications [14]. Therefore, strengthening prenatal diagnosis, genetic screening and other front-end screening is an important measure to prevent MPS II. According to a survey in 2018, 67.13% of the 1,010 adult rare disease patients in China were diagnosed in different places [15]. Similarly, MPS II patients had to transfer to several hospitals for diagnosis and treatment. This indicates that the overall diagnosis and treatment capacity of rare diseases is insufficient, resulting in rare diseases being mainly concentrated in large tertiary-level public hospitals and less involved in primary hospitals, which delays patient treatment to a certain extent [16]. It is recommended to establish a national rare disease diagnosis and treatment network and optimize the diagnosis and treatment process from screening to rehabilitation to ensure that patients receive continuous and seamless services.

The results of this study show that an increase in the number of hospitalizations, prolonged hospitalization days, and outpatient/emergency visits leads to a heavier financial burden for patients. Prolonged hospitalization days as well as prolonged hospitalizations increase the cost of hospitalization, including medications, tests, and possible additional accommodation costs. Similarly, an increase in the number of outpatient/emergency visits can add to the burden of outpatient, emergency, and treatment costs for patients. At the same time, the disease affects the work of patients and their families, further exacerbating financial stress. The results of this study showed that the cost of patients treated with HSCT was significantly higher than that of those who did not receive the treatment, due to the high complexity of the procedure [17], the cost of postoperative monitoring and management of complications, and the evaluation of the need for antirejection drugs. Currently, national and international guidelines and consensus unanimously recommend ERT as the standard treatment for MPS II, but it is expensive and requires long-term treatment. Despite insurance and assistance, patients’ out-of-pocket costs are still high, and most families give up treatment due to financial difficulties. Therefore, it is urgent to reduce the cost of treatment and optimize the reimbursement mechanism.

Currently, MPS II patients face a heavy financial burden, and most of the treatment costs still need to be paid by patients out of pocket, despite their participation in the national basic health insurance. This is mainly due to China’s limited health insurance funds and the need to improve the multiple protection methods. Compared with foreign countries that began to pay attention to the issue of rare diseases as early as the 1980s [18], China’s policy research in rare diseases has mainly focused on the last decade. In order to alleviate the economic pressure on patients and families, the government should gradually include rare diseases that have a greater impact on patients, such as MPS II, into the scope of basic medical insurance and medical assistance, forming a multi-level protection system. Through these measures, it is expected to alleviate the financial burden of MPS II patients, ensure that they can receive timely and effective treatment, and improve their quality of life and degree of recovery.

## Limitations

There is limited research on the disease burden of MPS II both domestic and international, and it is not possible to compare the results with other studies. The survey data were mainly derived from patients’ retrospectives, and the acquisition of retrospective data may be affected by the limitations of patients’ memories, subjective memory bias, and the passage of time, but they also provided valuable information for our study.

## Data Availability

The original contributions presented in the study are included in the article/supplementary material, further inquiries can be directed to the corresponding author.
